# Embroidered Electrode with Silver/Titanium Coating for Long-Term ECG Monitoring

**DOI:** 10.3390/s150101750

**Published:** 2015-01-15

**Authors:** Markus Weder, Dirk Hegemann, Martin Amberg, Markus Hess, Luciano F. Boesel, Roger Abächerli, Veronika R. Meyer, René M. Rossi

**Affiliations:** 1 Laboratory for Protection and Physiology, EMPA, Swiss Federal Laboratories for Materials Science and Technology, Lerchenfeldstrasse 5, CH-9014 St. Gallen, Switzerland; E-Mails: luciano.boesel@empa.ch (L.F.B.); veronika.meyer@empa.ch (V.R.M.); rene.rossi@empa.ch (R.M.R.); 2 Laboratory of Advanced Fibers, EMPA, Swiss Federal Laboratories for Materials Science and Technology, Lerchenfeldstrasse 5, CH-9014 St. Gallen, Switzerland; E-Mails: dirk.hegemann@empa.ch (D.H.); martin.amberg@empa.ch (M.A.); 3 Unico Swiss Tex GmbH, Chälengasse 4, CH-6053 Alpnachstad, Switzerland; E-Mail: info@unico-swiss-tex.ch; 4 Cardiovascular Research Institute, University Hospital Basel, Spitalstrasse 2, CH-4031 Basel, Switzerland; E-Mail: roger.abaecherli@usb.ch

**Keywords:** electrocardiogram, ECG, embroidered electrode, silver-titanium coated polyester electrode, long-term ECG monitoring, ECG belt

## Abstract

For the long-time monitoring of electrocardiograms, electrodes must be skin-friendly and non-irritating, but in addition they must deliver leads without artifacts even if the skin is dry and the body is moving. Today's adhesive conducting gel electrodes are not suitable for such applications. We have developed an embroidered textile electrode from polyethylene terephthalate yarn which is plasma-coated with silver for electrical conductivity and with an ultra-thin titanium layer on top for passivation. Two of these electrodes are embedded into a breast belt. They are moisturized with a very low amount of water vapor from an integrated reservoir. The combination of silver, titanium and water vapor results in an excellent electrode chemistry. With this belt the long-time monitoring of electrocardiography (ECG) is possible at rest as well as when the patient is moving.

## 1. Introduction

With the electrocardiogram (ECG), a multitude of important information about the state of health of the heart can be recorded, as well as the heart rate variability (HRV) [1] or the psycho-physiologic situation of a person. There is an increasing demand for electrodes allowing the long-time monitoring of the ECG, not only in hospitals, but also in the everyday life of patients (home-monitoring) [2]. Several devices for the continuous monitoring of the health of persons are available. The entire chain of data collection, data transfer by mobile phone or wireless local area networks (WLAN), and data processing in a medical center is established today [3]. However, high-quality sensor systems still remain a major challenge. The traditional gel electrodes with silver/silver chloride (Ag/AgCl) are the today's gold standard, *i.e.*, a benchmark that should be outperformed by new developments. For long-term monitoring, e.g., of heart patients, persons at stress risk, or burnout prevention, sensors are needed which can be worn on the body for several days. With gel electrodes the use is limited to one or maximum two days, since they tend to run dry and give rise to skin irritations on the long term. For a comprehensive review on the basics of ECG and the measurement of ECG with traditional gel electrodes the reader is referred to Xu *et al.* [4]. Electrodes for long-term monitoring must thus fulfil many requirements:

‐ biocompatibility (according to ISO 10993-10),
‐ no cytotoxicity (according to ISO 10993-5),
‐ constant signal quality, also with dry skin and moving body,
‐ low impedance, leading to low susceptibility to interference,
‐ possibility of reutilization,
‐ long-time durability,
‐ washing fastness,
‐ no extra skin preparation needed (such as removal of hairs and stratum corneum).

In recent years, new patch electrodes with integrated electronics were launched which can be adhered to the skin (e.g., ZIO^®^ XT by iRhythm Technolgies, San Francisco, CA, USA, or MultiSense™ by Rhythm Diagnostic Systems, Dover, DE, USA). Although these devices are very small, they have an important drawback as they are thicker than the usual Ag/AgCl electrodes.

Dry electrodes in chest belts are in widespread use and are mainly based on textile [4,5] or foam electrodes [6]. They yield good signals, even on dry skin, therefore they can also be used during low activity periods or during sleep. However, they are usually made from a conductive polymer which contains unhealthy chemical compounds such as monomers and isocyanates, therefore they should not be worn for any length of time. These belt systems can be used ubiquitously for many different situations, and they can be easily fitted to the physique of a person. However, they yield only the heart rate, and sometimes also the HRV, but no proper ECG.

Another approach is sensor-equipped underwear but it is difficult to adjust the needed pre-stressing force at the electrode for reliable data acquisition. Non-contact, capacitive electrodes are of special interest since they do not require an ohmic connection to the body [7,8]. These non-contact electrodes are integrated in the fabric and can be used without preparation [9]. However, the problem of the uncontrolled capacity change during physical activity has still to be solved. Nevertheless, capacitive electrodes are an interesting development; they can be built into a car seat and generate useful ECG data as long as the driver travels quietly on a highway [10]. The advantage of such systems is that capacitive electrodes are not worn on the body but that the ECG can be recorded through the clothes. However, as soon as the driver moves the upper body (e.g., during steering or shifting the gear), strong artifact signals are generated. Leicht *et al.* [11] built an ECG system with capacitive electrodes for a car seat with an integrated moisturization to reduce such artifacts.

In this paper we present embroidered textile electrodes prepared from a polyethylene terephthalate (PET) yarn which is coated with silver and a thin titanium topcoat. Two electrode patches are embroidered into a textile breast belt together with a small water reservoir which guarantees a minimal level of water vapor in the sensors (moisturizing). The combination of silver and titanium in a low-humidity environment was found to yield good and stable ECG signals. All materials used for the belt are skin-friendly and nonhazardous.

## 2. Development of the ECG Electrode and Belt

### 2.1. The Coated Fiber

We used a polyethylene terephthalate yarn of about 50 μm diameter (PET 150dtexf48z60 by Serge Ferrari Tersuisse AG, Emmenbrücke, Switzerland) as starting material and coated it with silver and titanium by plasma sputtering [12,13]. Specifically, the yarn was sputtered with a metallic silver coating of 100 nm thickness and then with a metallic titanium coating of 4–7 nm thickness, using a magnetron sputter apparatus [14]. As target materials, Ag and Ti, respectively (5 cm diameter, 19 cm^2^ area, 99.99% purity, supplied by Umicore AG (Balzers, Liechtenstein)) were used for continuous coating on the PET yarn. Both layers were applied in metallic mode in the same vacuum chamber. To obtain a uniform by-stacked layer structure along the fiber, the yarn was transported reel-to-reel first 40 times through the coating zone of the silver target and subsequently 40 times through the coating zone of the titanium target. The speed was varied between 5 and 10 m·min^−1^ in order to adjust the coating thickness. The sputtering was performed in a vacuum chamber at 2 Pa argon atmosphere using a 2′′ magnetron sputtering system (“stiletto” type ST20-O-C-M supplied by AJA International Inc., North Scituate, MA, USA).

### 2.2. The Embroidered Electrode

**Figure 1.**
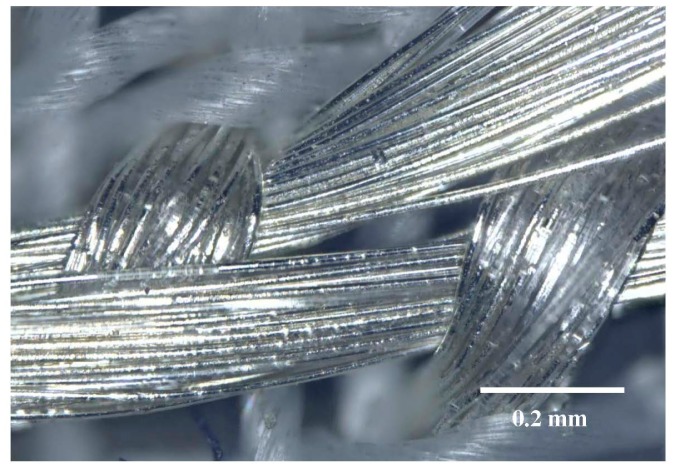
Detail of the embroidered electrode made from Ag/Ti-coated PET yarn.

Electrode pads of size 2 × 7 cm^2^ were directly embroidered into the fabric used for the ECG belt (see Section 2.4 below) as shown in Figure 1.

### 2.3. The Wetting Pad

**Figure 2.**
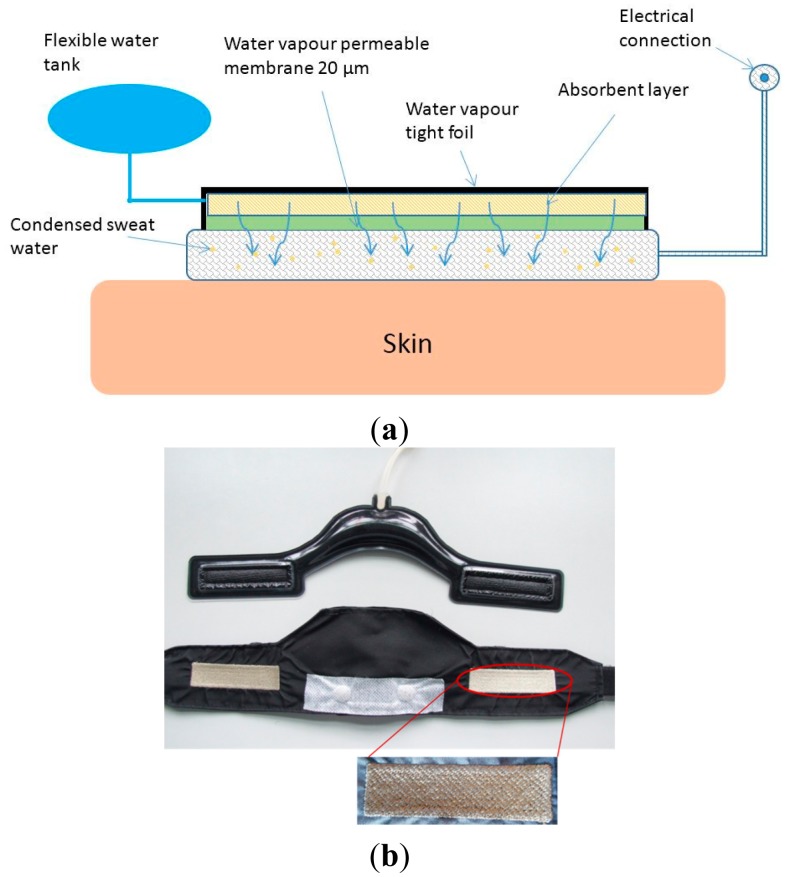
(**a**) Sketch of the wetting device; (**b**) Prototype of the wetting pad (**above**) and the ECG belt with embroidered electrodes (**below**).

As explained above, the embroidered electrodes with Ag/Ti coating need to be in a humid environment for good signal quality when the person is moving. For this reason, we developed a device to continuously deliver a very low amount of water vapor to the electrode. This is done by using a wetting pad made from polyester, see Figure 2 below [15].

Two pieces of Sympatex polyester membrane (20 μm thickness), welded by laser technology, form the evaporation areas. They are impermeable to liquid water but do allow the passage of water vapor. The filling volume of the pad is approx. 30 mL of water which is stored in a centered reservoir. The wetting pad is pouched into the ECG belt (see below) in order that the evaporation areas match the position of the electrodes in the belt. The permeability of the membrane was chosen to allow about 1–2 g of water evaporation per day, mainly due to the body heat of the wearer. After the permeation through the membrane, a high relative humidity in the electrode creates a favorable silver-titanium-water electrode chemistry. For good signal quality, even during movement, it is important that the amount of water in the electrode is very low, a prerequisite which is fulfilled with our device.

### 2.4. The ECG Electrode and Belt

The belt is made from polyester and an elastic band, see Figure 2. Two zones of Ag/Ti-coated PET yarn were directly embroidered into the fabric, forming the electrodes. With this technique, the yarn is exposed on both sides of the fabric, allowing an atmosphere with high relative water vapor pressure. The wetting pad fits into a pouch on the inner side of the belt, and the evaporation membranes are in close contact with the back side of the electrodes. The same coated yarn as used for the electrode is also used for an embroidered connection between the electrodes and the press-studs for the fixation of the data logger. With this set-up the logger can be easily removed for a hand-wash of the belt.

### 2.5. Data Acquisition and Processing

**Figure 3.**
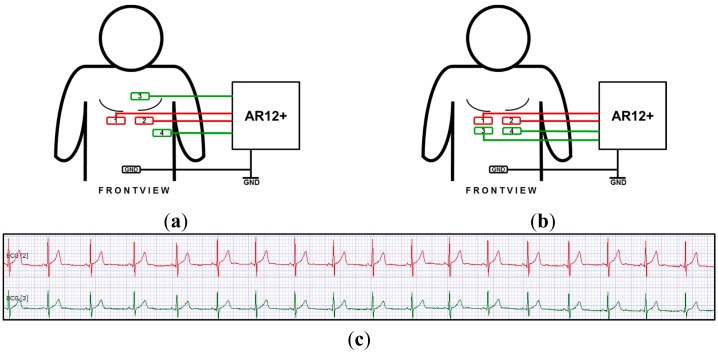
Positioning of the electrodes on the human thorax during our tests. (**a**) Moist textile electrodes placed horizontally within the belt (red #1 and #2); gel electrodes diagonally over the heart, *i.e.* the classical position for best signals (green #3 and #4); ground centered on the waist; (**b**) Gel electrodes placed as close to the belt as possible; (**c**) Signals obtained with arrangement (b); The test person was at rest with a pulse of 70.

Data acquisition was carried out by a commercial 3-canal logger with a sampling rate of 8 kHz per canal (AR12+, Schiller AG, Baar, Switzerland). The electrodes were connected with the logger by shielded cables. The data was evaluated by the commercial software Darwin (Schiller). During our experiments, more than a dozen test persons wore the ECG belt with the embroidered electrodes and two classical Ag/AgCl gel electrodes for comparison, see Figure 3. Some of these persons volunteered for numerous repeated measurement periods.

For a direct comparison of performance, the “red” electrodes should be placed at the same ideal spots on the body as the “green” ones. However, electrodes mounted on a belt are difficult to be applied at the typical thorax positions. Therefore most experiments were performed as shown in Figure 3a. In addition, we made also tests with an arrangement as shown in Figure 3b which resulted in a lower signal intensity of the gel electrodes. It is about 1.3 mV for the embroidered electrodes and 0.6 mV for the gel electrodes.

## 3. Results and Discussion

**Figure 4.**
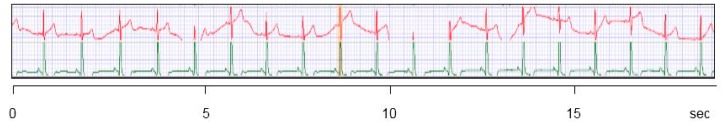
Electrode signals. **Red**: embroidered moist electrodes made from Ag-coated yarn worn with a chest belt; **Green**: classical Ag/AgCl gel electrodes. The test person wore the electrodes as shown in Figure 3a and was in light motion with a mean pulse of 60. For details see also the legend of Figure 5.

Table 1 presents the suitability of the investigated textile electrodes. A silver coating alone results in electrodes with limited application areas; especially, no usable ECG signals can be registered or the ECG signals are not stable. As shown in Figure 4, the baseline also changes strongly.

The oscillation of the baseline is approximately in the range of 0.8 mV. In addition, pure metallic silver in a low-humidity environment can locally cause high silver ion concentrations resulting in cytotoxic conditions when worn directly on the skin. Therefore, the silver coating was subsequently coated with a nanoscaled (metallic) titanium layer using the same sputtering equipment with a Ti target. The outermost layer oxidizes in contact with air which decreases the release of silver ions by about a factor of 100, and the electrode passes the ISO 10993-5 cytotoxicity test [16]. The titanium dioxide layer, approx. 2 nm thick, acts as an effective passivation while still enabling a low contact resistivity.

Dry Ag/Ti coated electrodes yield good signals at rest, and if they are humidified, they can also be used in motion (Table 1). Even large body motions, without perspiration, do not impair the signal quality or the baseline stability. The water vapor atmosphere prevents artifacts which otherwise result from the friction between electrode and skin.

**Figure 5.**
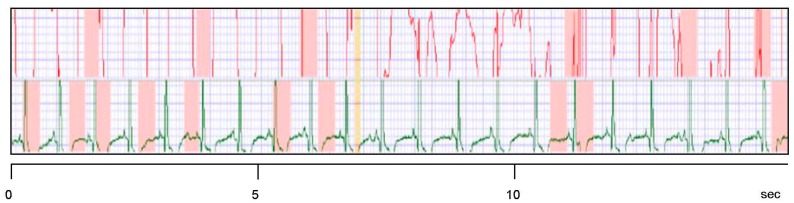
Example of “not suitable” signals (**red curve**) as obtained by embroidered Ag/Ti electrodes under dry conditions and in motion. The test person wore the electrodes as shown in Figure 3a and its mean pulse was 86.

**Figure 6.**
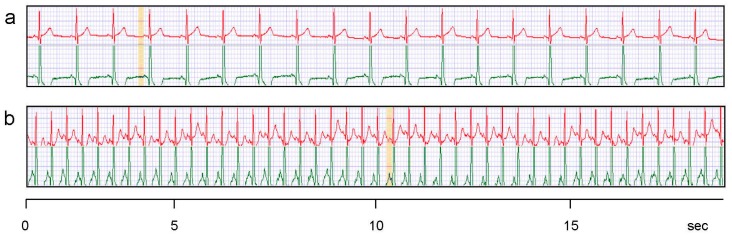
Electrode signals. **Red**: 2 embroidered moist electrodes with Ag/Ti coating worn with the belt; **Green**: 2 classical Ag/AgCl gel electrodes placed diagonally over the heart (Ambu Blue Sensor by Synmedic AG, Zurich, Switzerland). For details see Figure 3. (**a**) At rest (pulse 63); (**b**) In motion (pulse 146).

Although the classification “suitable” *vs.* “not suitable” might appear to be influenced by subjective evaluation, it is straightforward. A good ECG curve has (nearly) no artifacts and reveals the QRS complex as well as the T and P waves clearly, compare Figures 5 and 6 below. Signals as shown in Figure 4 (red curve) can only be used for pulse or HRV measurements due to the baseline instability. The case of “Ag/Ti coated dry” is presented in Figure 5 as an example of “not suitable”.

Figure 6 shows the ECG signals at rest and in motion which can be obtained with moist Ag/Ti electrodes. The other experimental set-ups mentioned in Table 1 gave poorer results or no signals at all. The embroidered electrodes yield signals which are comparable with the ones from Ag/AgCl gel electrodes. Although the results from the classical electrode and the embroidered one do not match, it is important to stress out the setup shown in Figure 3. The classical electrode was positioned directly across the heart, in the most favorable position for acquisition of ECG signals. Ours, on the other hand, were positioned below the heart, so we were already expecting a slightly different signal for both electrodes, what was confirmed as shown in Figure 5. This was unavoidable as it was our intention to compare the electrodes signals being used simultaneously by each test subject. For future developments also single electrodes shall be used which are located on a tight underwear to be in close contact to the body surface. Only body areas with a convex shape can guarantee a certain pressure of the electrodes on the skin.

The long-term stability of the embroidered electrode is excellent, even in motion. That is mainly due to the fact that their resistivity is in the range of a few ohms. The lower the ohmic resistance of an electrode, the more information can be obtained from the lead. (In contrast, the customary Ag/AgCl gel electrodes or Polar dry belt electrodes show a purely ohmic resistance of 4–5 kΩ.) The newly developed Ag/Ti electrodes have a very low conductance despite the nonconductive titanium oxide layer which does not impair the contact resistivity [17].

In recent years there has been a widespread interest on the development of textile-based electrodes [4,5,11]. Most of the reported works, however, use silver coatings on polymer fibers [4,5] which do not yield very good signals as we have demonstrated here. Another favored approach is the inclusion of stainless steel together with cotton or polyester fabrics as the electrode [4,5]. Moreover, the majority of those works use only dry electrodes. Our setup, therefore, is different than previous works in several aspects: our electrodes are embroidered with PES fiber coaxially coated with two metals (Ag/Ti). The nanoscaled coating is performed via plasma sputtering directly on the yarn [12]. The additional titanium layer enhances the signal quality while it decreases the cytotoxicity issues of silver. Finally, our sensor integrates a wetting device which releases only a minor amount of humidity through a water vapor permeable membrane which is facing the electrode from the rear side (Figure 2a) [18]. The water vapor which passes the membrane condenses on the embroidered pad. With this humidity, the electrode is much less influenced by movement artifacts and has a similar behavior like a person sweating in a liquid state. Humidification pads have also been the focus of another recent publication [11], where an approach similar to ours has been presented. Humidification of the textile electrode was found to improve the signal quality by a factor of 3.5. However, the described sensor setup is different from ours; it is intended as a non-contact electrode for short monitoring times (e.g., during driving) [11], whereas we concentrate on developing a sensor for long-term monitoring (several days). Nonetheless, their results are in agreement with ours.

Our results as shown in Figure 6 are a prerequisite for the long-term use as surveillance devices for patients or risk persons. The titanium coating which covers the silver layer is skin-friendly, and it decreases the transport of potentially hazardous silver ions to the skin. The coating is durable as was found during more than 100 experiments with volunteers. The water reservoir of the wetting pad is large enough for the continuous ECG registration over 5–10 days without a refill. First tests over such long time spans did not show skin irritations due to the titanium or the locally increased skin moisture.

## 4. Conclusions and Outlook

The results obtained with the embroidered, humidified Ag/Ti coated electrodes are very promising for long-term ECG recording. Thanks to their low impedance of a few ohms and the water vapor charging, they are insensitive to motion artefacts. The passivation by Ti allows long-term humid measuring conditions while avoiding cytotoxicity issues, combined with stable data recording. We intend to continue our research and development with regard to an improved plasma coating process of the fiber as well as the production of single electrodes.

Besides the surveillance of patients in a hospital or rehab institution, home-monitoring will gain importance in the future [2]. Long-term measurements of these electrodes showed the same signal quality and signal strength after 1 h as after 72 h of use. Risk persons can stay in their familiar surroundings while knowing that medical assistance is quickly available if needed—a real gain in quality of life. Wearable, embroidered electrodes also have a potential for the surveillance of professionals in risk jobs such as firefighters. Today it is an open question if ECG data acquisition and transfer will also be used in sports or in everyday life, e.g., as an instrument which supports people in their motivation for more exercise. On the other hand, this type of electrode is also usable for active muscle stimulation or for interference wave therapy of the colon.

In summary, we claim that the new embroidered electrodes with Ag/Ti coating fulfills the eight requirements listed in the Introduction with regards to biocompatibility, cytotoxicity, signal stability, impedance, reusability, durability, washing fastness, and ease of use. The combination of all these properties will lead to a product which is also economically feasible. As future work we intend to check the reliability of the embroidered electrodes, as worn in the ECG belt, with a larger panel of people and in everyday use.

## Figures and Tables

**Table 1.** Signal quality and application areas of the fiber electrodes.

**Type of PET Yarn Electrode**	**Signal Quality**		**Application Area**			

			**HRV Measurement** *		**ECG Diagnostics**	

	**At Rest**	**In Motion**	**At Rest**	**In Motion**	**At Rest**	**In Motion**
Ag coated dry	signal OK, baseline fluctuations	no signal	**suitable**	not suitable	not suitable	not suitable
Ag coated moist	signal OK, baseline fluctuations	signal OK, baseline fluctuations	**suitable**	**suitable**	not suitable	not suitable
Ag/Ti coated dry	excellent	no signal	**suitable**	not suitable	**suitable**	not suitable
Ag/Ti coated moist	excellent	excellent	**suitable**	**suitable**	**suitable**	**suitable**

* Analysis of the heart rate variability by detection of the RR peaks in the ECG.

## References

[1] Isler Y., Kuntalp M. (2007). Combining classical HRV indices with wavelet entropy measures improves to performance in diagnosing congestive heart failure. Comput. Biol. Med..

[2] Fergus P., Iram S., al-Jumeily D., Randles M., Attwood A. (2012). Home-based health monitoring and measurement for personalised healthcare. J. Med. Imaging Health Inform..

[3] Chi Y.M., Cauwenberghs G. Wireless non-contact EEG/ECG electrodes for body sensor networks.

[4] Xu P.J., Zhang H., Tao X.M. (2008). Textile-structured electrodes for electrocardiogram. Text. Prog..

[5] Beckmann L., Neuhaus C., Medrano G., Jungbecker N., Walter M., Gries T., Leonhardt S. (2010). Characterization of textile electrodes and conductors using standardized measurement setups. Physiol. Meas..

[6] Lin C.T., Liao L.D., Liu Y.H., Wang I.J., Lin B.S., Chang J.Y. (2011). Novel dry polymer foam electrodes for long-term EEG measurement. IEEE Trans. Biomed. Eng..

[7] Wartzek T., Brüser C., Walter M., Leonhardt S. (2014). Robust sensor fusion of unobtrusively measured heart rate. IEEE J. Biomed. Health Inform..

[8] Spinelli E., Haberman M., García P., Guerrero F. (2012). A capacitive electrode with fast recovery feature. Physiol. Meas..

[9] Lin Y.D., Chien Y.H., Wang S.F., Tsai C.L., Chang H.H., Lin K.P. (2013). Implementation of multiple-channel capacitive ECG measurement based on conductive fabric. Biomed. Eng. Appl. Basis Comm..

[10] Heuer S. (2011). Ambiente Kapazitive EKG-Messung—Elektroden, Systeme und Konzepte. Ph.D. Thesis.

[11] Leicht L., Eilebrecht B., Weyer S., Wartzek T., Leonhardt S. (2014). Active humidification for capacitive-resistive ECG—Systems. Biomed. Technol..

[12] Hegemann D., Amberg M., Ritter A., Heuberger M. (2009). Recent developments in Ag metallised textiles using plasma sputtering. Mater. Technol..

[13] Amberg M., Grieder K., Barbadoro P., Heuberger M., Hegemann D. (2008). Electromechanical behavior of nanoscale silver coatings on PET fibers. Plasma Process. Polym..

[14] Amberg M., Geerk J., Keller M., Fischer A. (2004). Design, characterisation and operation of an inverted cylindrical magnetron for metal deposition. Plasma Devices Oper..

[15] Haag A., Weder M., Camenzind M. Adjustment of water vapor resistance using laser transmission welding. Tech. Text..

[16] Amberg M., Rupper P., Storchenegger R., Weder M., Hegemann D. Controlling the release from silver electrodes by titanium adlayers for health monitoring. NanoMed.

[17] Oehzelt M., Koch N., Heimel G. (2014). Organic semiconductor density of states controls the energy level alignment at electrode interfaces. Nat. Comm..

[18] Weder M. (2013). Moistened Sensor Contact Unit.

